# Left ventricular twist mechanics during incremental cycling and knee extension exercise in healthy men

**DOI:** 10.1007/s00421-016-3506-8

**Published:** 2016-12-05

**Authors:** Alexander Beaumont, John Hough, Nicholas Sculthorpe, Joanna Richards

**Affiliations:** 1 School of Science and Sport, Institute of Clinical Exercise Physiology and Health Science, University of the West of Scotland, Glasgow, UK; 2 Department of Sport Science and Physical Activity, Institute of Sport and Physical Activity Research, University of Bedfordshire, Bedford, UK

**Keywords:** Exercise, Rotation, Left ventricle, Speckle tracking echocardiography

## Abstract

**Purpose:**

The objective of the present study was to investigate left ventricular (LV) twist mechanics in response to incremental cycling and isometric knee extension exercises.

**Methods:**

Twenty-six healthy male participants (age = 30.42 ± 6.17 years) were used to study peak twist mechanics at rest and during incremental semi-supine cycling at 30 and 60% work rate maximum (*W*_max_) and during short duration (15 s contractions) isometric knee extension at 40 and 75% maximum voluntary contraction (MVC), using two-dimensional speckle tracking echocardiography.

**Results:**

Data presented as mean ± standard deviation or median (interquartile range). LV twist increased from rest to 30% *W*_max_ (13.21° ± 4.63° to 20.04° ± 4.76°, *p* < 0.001) then remained unchanged. LV systolic and diastolic twisting velocities progressively increased with exercise intensity during cycling from rest to 60% *W*_max_ (twisting, 88.21° ± 20.51° to 209.05° ± 34.56° s^−1^, *p* < 0.0001; untwisting, −93.90 (29.62)° to −267.31 (104.30)° s^−1^, *p* < 0.0001). During the knee extension exercise, LV twist remained unchanged with progressive intensity (rest 13.40° ± 4.80° to 75% MVC 16.77° ± 5.54°, *p* > 0.05), whilst twisting velocity increased (rest 89.15° ± 21.77° s^−1^ to 75% MVC 124.32° ± 34.89° s^−1^, *p* < 0.01). Untwisting velocity remained unchanged from rest [−90.60 (27.19)° s^−1^] to 40% MVC (*p* > 0.05) then increased from 40 to 75% MVC [−98.44 (43.54)° s^−1^ to −138.42 (73.29)° s^−1^, *p* < 0.01]. Apical rotations and rotational velocities were greater than basal during all conditions and intensities (all *p* < 0.01).

**Conclusion:**

Cycling increased LV twist to 30% *W*_max_ which then remained unchanged thereafter, whereas twisting velocities showed further increases to greater intensities. A novel finding is that LV twist was unaffected by incremental knee extension, yet systolic and diastolic twisting velocities augmented with isometric exercise.

## Introduction

One of the first observations that the heart permits a rotary motion during contraction dates from the seventeenth century where Lower described twisting mechanics as, “… the wringing of a linen cloth to squeeze out the water …” (Lower 1669). The wringing motion identified by Lower is due to the myocardium comprising a single band of myocardial tissue, wound upon it forming a helical ventricular band and a circumferential loop encompassing the left and right ventricular cavities; a configuration referred to as the Torrent-Guasp model (Biswas et al. 2013). A continuum of fibre orientation (Streeter et al. 1969) and a complex interaction between the helical and circular fibres determines the speed and degree of the wringing motion of the left ventricle (LV) (Mor-Avi et al. 2011). This twisting action has been quantifiably assessed using speckle tracking echocardiography (STE) over the last decade. ‘LV twist mechanics’ describe systolic twisting and diastolic untwisting due to the opposing rotations of the base and apex (Sengupta et al. 2008).

Early experiments using animal models (Dong et al. 1999; Gibbons Kroeker et al. 1995) and observations in human studies (Burns et al. 2010; Weiner et al. 2010b) indicate LV twist mechanics are sensitive to physiological changes in cardiac loading. Likewise, the influence of positive inotropic stimulation on twist mechanics is also well documented (Notomi et al. 2008). Twisting mechanics of the LV play a fundamental role in enhancing cardiac function following the transition from rest to exercise (Drury et al. 2012). Additional potential energy stored during systolic twisting results in greater untwisting to facilitate an intraventricular pressure gradient and accelerate passive diastolic filling via increased suction to draw blood toward the apex (Notomi et al. 2006). Twist mechanics increase during dynamic submaximal exercise in the healthy heart (Lee et al. 2012; Stohr et al. 2012), however, only two studies have assessed twist during acute incremental exercise using STE (Doucende et al. 2010; Stohr et al. 2011) both demonstrating twist mechanics progressively increased, indicative of systolic-diastolic coupling. However, to the best of our knowledge, Stohr et al. (2011) is the only study to date having investigated twist mechanics beyond 40% peak power, showing a plateau at ~50% of peak power.

Data regarding twist mechanics during exercise modes which specifically elevate afterload are sparse. Two studies using sustained (3 min) isometric hand grip (IHG) at 40% maximal voluntary contraction (MVC) (Balmain et al. 2016; Weiner et al. 2012) and reported decreased LV twist. No study to date has examined twist mechanics during incremental isometric exercise at or beyond 40% MVC, therefore LV function, assessed by twist mechanics, remains unknown during more intense exercise. Although IHG is a feasible method of inducing afterload (Weiner et al. 2012), the muscle mass involved is small and not a representative of isometric or dynamic resistance exercise. Consequently, twist mechanics during lower body, short duration isometric exercise has not been studied to date. Given that the blood pressure responses during isometric knee extension exercise are reportedly greater than that of IHG (Smolander et al. 1998), this may be a more ecologically valid model to assess the LV response to resistance exercise.

No study to date has investigated the twist mechanics in response to dynamic and resistance exercises in the same cohort. Therefore, we sought to investigate twist mechanics during incremental cycling and lower body isometric exercise in a healthy population. We hypothesised that LV twist mechanics would, (1) plateau during the submaximal exercise and (2) progressively decrease during the isometric exercise.

## Materials and methods

### Study design and population

Twenty-seven healthy males (18–40 years) were recruited for this cross-sectional study. A medical questionnaire was used to exclude a past history or known current diagnosis of coronary heart disease, hypertension, diabetes mellitus, myocardial infarction, peripheral artery disease or sudden cardiac death in immediate family members. Participants were required to avoid vigorous physical activity, and consumption of alcohol (24 h) and caffeine (12 h) prior to data collection. Before initiation of this study, the University of Bedfordshire ethics committee reviewed and approved the protocol which was conducted in accordance with the declaration of Helsinki.

### Protocol and experimental procedures

Participants attended the University of Bedfordshire’s Sport Science Laboratories on two separate occasions at the same time of day. Each visit was separated by at least 24 h but <7 days. Demographic information, physiological assessment and a baseline (resting) echocardiographic assessment were collected/completed on the first visit to the laboratory. Following this collection of data, during visit 2 each participant completed submaximal cycling and isometric knee extension protocols to obtain twist mechanics data during exercise.

With participants unshod and in minimal clothing height (HAR-98.602, Harpenden, Holtain Ltd, Crymych) and body mass (BWBO800, Tanita, Netherlands) were recorded and used to calculate body surface area, with the Mosteller formula (Mosteller 1987). After a 5 min rest in the supine position, heart rate (HR) (FS1, Polar Electro Oy, Kempele, Finland), systolic (SBP) and diastolic blood pressures (DBP), using manual sphygmomanometry, were recorded and then used to calculate mean arterial pressure (MAP) as Weippert et al. (2013):

$${\text{MAP}} = \frac{{({\text{SBP}} + 2 \times {\text{DBP}})}}{3}.$$

After a baseline echocardiographic examination, participants completed an incremental exercise test to exhaustion on a dedicated semi-supine ergometer (eBike-L, ergoline GmbH, GE Healthcare) using breath-by-breath analysis of expired gases (Metalyser 3B, Cortex, Germany). The following criteria were used to establish obtainment of maximal oxygen uptake in accordance with the British Association of Sports and Exercise Sciences (BASES) (Cooke 2009): (1) a plateau in oxygen uptake (${\dot{\text{V}}\text{O}}_{2}$), (2) a respiratory exchange ration ≥1.15, (3) HR within ±10 beats min^−1^ of age predicted maximum (220-age), (4) subjective fatigue and volitional exhaustion, and (5) rating of perceived exertion ≥19 according to the Borg scale. Since a plateau in ${\dot{\text{V}}\text{O}}_{2}$ was absent in multiple participants, peak oxygen consumption (${\dot{\text{V}}\text{O}}_{\text{peak}}$) was reported and confirmed with the attainment of two of the five above criteria. Work rate maximum (*W*_max_) was calculated using the equation (Kuipers et al. 1985):

$$W_{ \hbox{max} } = W_{\text{com}} + \left( {\frac{t}{60}} \right) \times W,$$

where ‘*W*_com_’ refers to the last completed stage, ‘*t*’ the number of seconds in final stage completed and ‘*W*’ the final load increment (Arts et al. 1993). For relative workloads, 30 and 60% *W*_max_ were calculated for use in the submaximal protocol.

#### STE during cycle ergometry

During visit 2, participants undertook a submaximal bout of cycling for echocardiographic assessment. Following a 10 min rest on the semi-supine ergometer (eBike-L, ergoline GmbH, GE Healthcare), participants performed incremental exercise of 2 × 5 min stages at 30 and 60% *W*_max_, where the last 3 min of each stage was used for echocardiographic data collection (Stohr et al. 2012). During the data collection period, a cadence of 60 rpm was maintained to limit upper body oscillation, with HR using a pulse oximeter (9590, Nonin Medical, Netherlands), and manual blood pressures recorded at the end of each stage.

#### STE during isometric knee extension

Following 10 min seated rest, participants then lay supine onto an isokinetic dynamometer (Kin-Com 125E Plus, Chattecx Corporation, Chattanooga, USA) to conduct all isometric contractions. Participants undertook an isometric contraction of the quadriceps with the knee extension angle fixed at 130°. The participant’s dominant leg was used with the joint centre aligned with the axis rotation point of the crank arm (Kong and van Haselern 2010), an ankle pad connected to the load cell strapped proximal to the malleoli with a thigh and hip strap to prevent unnecessary limb movement while avoiding restricted blood flow (Marginson and Eston 2001). Participants were instructed to keep their back flat and generate force from the quadriceps. A warm-up of 5–10 submaximal contractions was performed prior to the MVC assessment, determined as the greatest of three maximal contractions (Adler et al. 2008). Each maximal effort was 5 s in duration with a 1 s transition period from rest and each separated by 2 min passive recovery (Bojsen-Moller et al. 2005). Following a 5 min rest, participants completed an incremental protocol consisting 2 × 15 s isometric contractions, separated by 2 min passive recovery, at intensities corresponding to 40 and 75% MVC to obtain a short-axis apical and basal images at both intensities. Pilot work found 15 s was the shortest contraction time possible, whilst still able to obtain SBP and DBP measurements. An upper intensity of 75% MVC was chosen based on previous findings of an unavoidable Valsalva manoeuvre at intensities ≥80% MVC (MacDougall et al. 1992). Participants were verbally instructed to breathe normally throughout the contraction, and refrain from holding their breath to avoid the performance of a Valsalva manoeuvre and participants were also reminded throughout the exercise to breath normally. A visual digital display was provided for participants to maintain the given intensities. Five cardiac cycles were recorded at the end of each contraction, whilst HR and manual blood pressures were obtained at the termination of contraction.

### Echocardiography

All participants underwent two-dimensional transthoracic echocardiographic examinations at rest in the left lateral decubitus position and during both exercise modalities using commercially available ultrasound equipment (Vivid 7, GE Medical, London) with a phased array transducer (3S 1.4–3.8 MHz). All image acquisition and measurement procedures as described below were conducted by the same investigator in accordance with established guidelines (Lang et al. 2015; Mor-Avi et al. 2011; Nagueh et al. 2009). Five cardiac cycles were obtained at end-expiration and analysed from a minimum of two consecutive cycles when three were not available.

#### Conventional structure and function

Standard parameters of LV structure and function were assessed only at baseline (rest) from previously saved images. The parasternal long axis view was used to determine LV internal measures, then used in the calculation of relative wall thickness and LV mass, in accordance with the American Society of Echocardiography and scaled to body surface area to obtain LV mass index (Lang et al. 2015). The apical four and two-chamber views enabled the generation of LV end-diastolic and end-systolic volumes using Simpson’s Biplane method and conventional systolic function, stroke volume and ejection fraction. Cardiac output was determined as the product of HR and stroke volume. Standard diastolic function was assessed using the apical four-chamber view for mitral inflow velocities during early and late diastolic filling, their ratio and deceleration time. Further, using pulsed-wave Doppler, lateral and septal tissue velocities were obtained at the mitral annulus during systole, early diastole and atrial systole from a spectral trace.

#### Speckle tracking derived twisting mechanics

Twist mechanics were measured at rest and during both submaximal cycling and isometric knee extension protocols. To account for tachycardia and prevent potential under sampling, image depth and sector width were adjusted to alter frame rate (range 70–80 frames per second), so temporal resolution was maximised but the ensuing reductions in spatial resolution were minimised. The frame rates concur with past work having used STE during submaximal dynamic cycling (Donal et al. 2011; Doucende et al. 2010; Lee et al. 2012) and IHG (Balmain et al. 2016; Stefani et al. 2008; Weiner et al. 2012). Frame rates remained constant within subjects across all conditions. The parasternal short-axis views were used to acquire basal and apical imaging planes. The basal level was determined as the highest imaging plane at which full myocardial thickness was present with the observation of surrounding mitral valve at end-systole and positioned as circular as possible with no visible papillary muscles (Weiner et al. 2010a). Given that apical rotation shows proportional increases with progressive caudal transducer movement (Stewart et al. 2016), apical images were captured proximal to the end-systolic luminal obliteration of the LV cavity with as much accuracy as possible (van Dalen et al. 2008). When an insufficient apical image was found following distal transducer movement, a second method of obtaining a ‘true’ apical view was utilised. Beginning in the apical four-chamber view, the transducer was then tilted upwards with attention paid to ensuring the lumen was as circular as possible before image capture (Stohr et al. 2016).

Images were analysed offline using semiautomated software (EchoPac software, GE Healthcare, UK) by one investigator. The endocardial border was manually detected and initially traced prior to automatic software tracking. The region of interest was manually adjusted until the epicardial border was correctly aligned to encompass the entire LV wall thickness whilst avoiding the echogenic pericardium (Mor-Avi et al. 2011). Data files were imported into an Excel spreadsheet (Microsoft Corporation, Washington, USA) where a cubic spline add-in (SRS1 Software, Boston, USA) was implemented to generate 300 data points for both systole and diastole. Due to variances in HR, rotations were normalised to 5% increments of systolic and diastolic cycles with the pre-defined aortic valve closure indicating end-systole (100% systole). Peak LV twist during ejection was determined as the difference between basal and apical rotations, expressed in degrees (°) (Santoro et al. 2014a); whilst peak untwisting velocity (PUV) was considered the largest negative deflection following peak twisting velocity (PTV) (Stohr et al. 2011). Time-to-peak untwisting velocity and the percentage of diastole that PUV occurred (%diastole) were also recorded.

### Intra-observer reproducibility

In a separate cohort comprising nine participants, within-day intra-observer reproducibility was assessed using coefficient of variation and determined for rotational parameters: apical rotation 3.1%, basal rotation 1.5%, twist 3.6%, PTV 28.6% and PUV 25.1%.

### Statistical analysis

Data presented as mean ± standard deviation or median (interquartile range). Normality of data distribution was assessed by Shapiro–Wilk. For normally distributed data, haemodynamic measures and twisting mechanics assessed during exercise were compared using one-way repeated measures analysis of variance (ANOVA) with post hoc Bonferroni correction. For non-normally distributed data, a Friedman test was employed followed by Wilcoxon signed-rank test. Apical and basal rotations were compared using *t* tests. All analyses were conducted using SPSS (V.21; IBM Company, SPSS Inc., Chicago, USA) and statistical significance granted at *p* < 0.05.

## Results

### Participant numbers and echocardiographic image quality

#### Number of participants within study

All participants completed the full protocol and echocardiographic data was determined in all participants at rest. However, cardiac images during all exercise conditions could not be obtained for one participant leading to exclusion from the study, resulting in a total sample of twenty-six participants available for statistical analyses. Furthermore, due to poor image quality, twisting mechanics data were not acquired during cycling at 30% *W*_max_ (*n* = 2) and 60% *W*_max_ (*n* = 3) and during knee extension at 75% MVC (*n* = 3). Consequently, an equal number of participants were required to determine net twist mechanics from apical and basal rotations which led to 22 and 23 participants during cycling and knee extension, respectively. A full data set was available for HR during both types of exercises (*n* = 26). An inability to obtain SBP and DBP during both intensities of cycling and knee extension (*n* = 1) and DBP at 75% MVC (*n* = 1), enabled 25 participants for SBP, DBP and MAP during cycling, whilst during knee extension 25 were available for SBP and 24 for DBP and MAP. Baseline participant characteristics and haemodynamic measures for the full cohort are presented in Table 1 and conventional echocardiographic structure and function shown in Table 2 (both *n* = 26).

**Table 1:** Participants characteristics and baseline haemodynamic measures

Standard demographics (*n* = 26)	
Age (years)	30.42 ± 6.17
Height (m)	1.77 ± 0.08
Mass (kg)	75.87 ± 9.90
BSA (m^2^)	1.93 ± 0.13
HR (beats min^−1^)	59.08 ± 11.95
SBP (mmHg)	118.00 ± 8.99
DBP (mmHg)	74.88 ± 5.73
MAP (mmHg)	89.26 ± 6.19

*HR* heart rate, *SBP* systolic blood pressure, *DBP* diastolic blood pressure, *BSA* body surface area, *MAP * mean arterial pressure

**Table 2:** Echocardiographic left ventricular structure and conventional systolic and diastolic function

*Structure*	
IVSd (mm)	10.23 ± 1.17
IVSs (mm)	13.08 ± 2.42
LVIDd (mm)	53.39 ± 3.85
LVIDs (mm)	36.42 ± 3.30
PWTd (mm)	9.75 ± 1.57
PWTs (mm)	15.15 ± 2.47
LVEDV (mL)	125.87 ± 26.18
LVESV (mL)	52.19 ± 12.27
LVM (g)	204.16 ± 37.04
LVMi (g/m^2^)	106.16 ± 19.77
RWT	0.37 ± 0.06
*Systolic function*	
EF (%)	58.39 ± 5.36
SV (mL)	73.68 ± 17.04
${\dot{\text{Q}}}$ (L min^−1^)	4.22 ± 0.76
Lateral S′ (cm s^−1^)	12.92 ± 1.94
Septal S′ (cm s^−1^)	9.23 ± 1.14
*Diastolic function*	
E-wave cm s^−1^)	72.23 ± 9.57
A-wave (cm s^−1^)	37.19 ± 7.33
*E/A* ratio	2.03 ± 0.51
DT (ms)	259.82 ± 62.94
Lateral E′ (cm s^−1^)	18.54 ± 3.00
Septal E′ (cm s^−1^)	12.46 ± 1.98
Lateral A′ (cm s^−1^)	7.92 ± 1.87
Septal A′ (cm s^−1^)	7.96 ± 1.61

Data are mean ± standard deviation

*d* end-diastole, *s* end-systole, *IVS* interventricular septum, *LVID* left ventricular internal diameter, *PWT* posterior wall thickness, *LVEDV* left ventricular end-diastolic volume, *LVESV* left ventricular end-systolic volume, *LVM* left ventricular mass, *LVMi* left ventricular mass index, *RWT* relative wall thickness, *EF* ejection fraction, *SV* stroke volume, $\dot{Q}$ cardiac output, *S*′ tissue Doppler systolic velocity, *E* mitral inflow early diastolic velocity, *A* mitral inflow late diastolic velocity, *E*/*A* ratio between early and late diastolic mitral inflow velocities, *DT* deceleration time, *E*′ tissue Doppler early diastolic velocity, *A*′ tissue Doppler late diastolic velocity

#### Quality of echocardiographic images during exercise

To establish the quality of images gathered during cycling and knee extension exercises, a grading system was employed by the sonographer rating images 1–4 (1 = excellent—all endocardial and epicardial boarders clearly defined and successful tracking of all segments; 2 = good—fully visible wall with successful tracking of at least five segments but image considered ‘hazy’; 3 = poor—areas of image undefined, but successful tracking of at least four out of six segments; 4 = no image available or unsuccessful tracking). Images were considered excellent good quality from 65% during cycling at 30% *W*_max_ and knee extension at 40 and 75% MVC, whilst 54% of images at 60% *W*_max_.

### Maximal physiological and haemodynamic parameters

Following the maximal incremental exercise test and MVC, group physiological parameters were: ${\dot{\text{V}}\text{O}}_{\text{peak}}$ (*n* = 25) 46.1 ± 12.5 mL kg^−1^ min^−1^, *W*_max_ 277 ± 49W; 30% *W*_max_ 83 ± 15 W; 60% *W*_max_, 166 ± 29W; MVC, 1103 ± 267N; 40% MVC, 441 ± 107N, and 75% MVC, 828 ± 200N. All haemodynamic data during cycling and knee extension are presented in Table 3. HR, SBP, DBP and MAP significantly (all *p* < 0.01) increased with progressive exercise intensity from baseline during both cycling and knee extension exercises.

**Table 3:** Haemodynamic measures at baseline and during both intensities of the cycling and isometric knee extension exercises

Variable	Baseline	Cycling (%*W*_max_)		Baseline	Knee extension (%MVC)	
		30%	60%		40%	75%
HR (beats min^−1^)	57.00 (14.00)	95.50 (10.75) †	127.50 (14.25)†‡	57.00 (14.00)	81.00 (23.75)†	96.50 (29)†‡
SBP (mmHg)^a^	118.28 ± 9.06	140.72 ± 9.31†	172.04 ± 20.86†‡	118.28 ± 9.06	130.08 ± 10.34†	146.40 ± 18.09†‡
DBP (mmHg)^b,c^	77.00 (9.50)	81.00 (3.50)*	82.00 (5.50)†‡	76.50 (8.75)	79.00 (8.50)*	82.00 (8.75)†‡
MAP (mmHg)^b,c^	89.32 ± 6.31	100.83 ± 5.40†	113.56 ± 7.91†‡	89.34 (10.59)	94.33 (5.40)†	103.83 (10.09)†‡

Data presented as mean ± standard deviation, non-normally distributed data presented as median (interquartile range)

*HR* heart rate, *SBP* systolic blood pressure, *DBP* diastolic blood pressure, *MAP* mean arterial pressure, *W*_*max*_ work rate maximum, *MVC* maximal voluntary isometric contraction

* *p* < 0.01 compared to baseline, † *p* < 0.0001 compared to baseline, ‡ *p* < 0.0001 compared to previous intensity

^a^ *n* = 25 during cycling and knee extension

^b^ *n* = 25 during cycling

^c^ *n* = 24 during knee extension

### LV twist mechanics

**Fig. 1:**
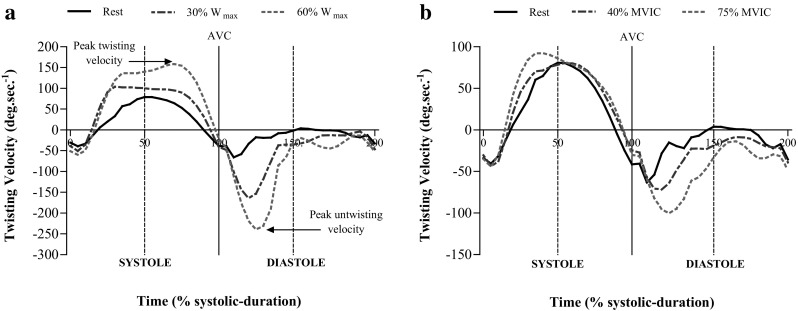
Torsion/time curves of twisting velocity throughout the cardiac cycle normalised to percentage of systolic duration (systole 0–100%, diastole 105–200%) in 5% increments from 22 participants during incremental cycling (**a**) and 23 participants during incremental knee extension (**b**). Data presented as means with *error bars* omitted for clarity. *AVC* aortic valve closure

All LV systolic and diastolic mechanics data are peak measures and presented in Table 4. Examples of torsion (twisting)/time curves at rest and intensities of cycling and knee extension exercises are shown in Fig. 1a, b.

**Table 4:** Peak systolic and diastolic LV rotations and rotational velocities during incremental cycling and isometric knee extension exercise

Variable	Cycling (%*W*_max_) (*n* = 22)			Knee extension (%MVC) (*n* = 23)		
	Baseline	30%	60%	Baseline	40%	75%
Systole						
Apical rot. (°)	9.40 ± 3.02	14.29 ± 4.20†	15.85 ± 5.04†	9.22 (5.55)	11.53 (6.30)*	13.58 (7.11)*
Basal rot. (°)	−4.32 ± 2.85	−6.40 ± 2.77*	−6.90 ± 2.57†	−4.56 ± 2.68	−3.26 ± 2.32	−4.73 ± 3.06
Twist (°)	13.21 ± 4.63	20.04 ± 4.76†	22.50 ± 4.93†	13.40 ± 4.80	14.72 ± 4.38	16.77 ± 5.54
Apical rot. velocity (° s^−1^)	62.12 (30.77)	123.32 (53.39) †	182.55 (42.82)†‡	59.65 (31.23)	100.95 (48.71)†	105.09 (70.98)†
Basal rot. velocity (° s^−1^)	−46.18 ± 15.28	−79.83 ± 25.51†	−114.63 ± 30.21†‡	−46.42 ± 14.95	−55.13 ± 15.34	−64.68 ± 19.84†
Twisting velocity (° s^−1^)	88.21 ± 20.51	145.11 ± 27.08†	209.05 ± 34.56†‡	89.15 ± 21.77	106.66 ± 21.00*	124.32 ± 34.89†**
Diastole						
Apical rot. velocity (° s^−1^)	−63.59 ± 17.05	−146.60 ± 31.52†	−204.35 ± 68.77†‡	−63.21 (25.81)	−80.15 (47.50)*	−119.03 (66.78)†‡
Basal rot. velocity (° s^−1^)	41.47 (25.94)	73.17 (48.76)†	109.41 (53.36)†‡	42.37 (26.22)	46.17 (16.68)	62.72 (28.00)
Untwisting velocity (° s^−1^)	−93.90 (29.62)	−193.19 (64.32)†	−267.31 (104.30)†‡	−90.60 (27.19)	−98.44 (43.54)	−138.42 (73.29)†‡
Time-to-peak (ms)	59.50 (28.50)	57.50 (27.00)	53.50 (28.00)	62.00 (31.00)	99.00 (64.00)	75.00 (75.00)
PUV % of diastole	7.00 (4.31)	15.45 (10.12)†	21.78 (12.53)†‡	8.33 (4.66)	19.11 (19.56)†	20.33 (30.11)†

Data presented as mean ± standard deviation, non-normally distributed data presented as median (interquartile range)

*W*_*max*_ work rate maximum, *MVC* maximum voluntary isometric contraction, *rot*. rotation, *PUV* peak untwisting velocity

* *p* < 0.05 compared to baseline, ** *p* < 0.05 compared to previous intensity, † *p* < 0.01 compared to baseline, ‡ *p* < 0.01 compared to previous intensity

#### Semi-supine cycling exercise

##### Systolic

**Fig. 2:**
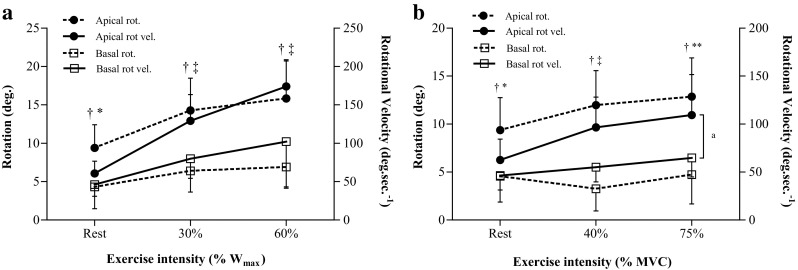
Peak systolic apical and basal rotations and rotational velocities from 22 participants during incremental cycling (**a**) and 23 participants during incremental knee extension (**b**). Statistical analyses performed using positive values. For clarity data presented as *positive values* expressed as mean ± standard deviation. *rot*. rotation, *rot* *vel.* rotational velocity, *W*_*max*_ work rate maximum, *MVC* maximum voluntary contraction. (*Superscript letter a*) Non-parametric statistical analysis for rotational velocity, *p < 0.01 between apical and basal rotational velocity, **p < 0.001 between apical and basal rotational velocity. ‡*p* < 0.0001 between apical and basal rotational velocity, †*p* < 0.0001 between apical and basal rotation

The cycling protocol resulted in significant increases in apical rotation, basal rotation and twist from baseline to 30% *W*_max_ which then remained unchanged to 60% *W*_max_. LV apical, basal rotation velocity and PTV progressively increased with exercise from rest to 60% *W*_max_. Systolic apical rotations and rotational velocities were significantly greater than the corresponding basal parameters at all intensities (Fig. 2a).

##### Diastolic

During cycling, peak diastolic apical, basal rotational velocities and PUV significantly increased with progressive exercise intensity from baseline to 60% *W*_max_. Time-to-peak untwisting velocity did not change between intensities, whereas PUV occurred significantly later into diastole, expressed as a %diastole with progressive intensity. During the cycling, peak apical untwisting velocity was significantly greater than basal untwisting velocity at all intensities (analyses performed on positive values) (rest, 63.59° ± 17.05° versus 48.32 ± 25.14° s^−1^, *p* < 0.05; 30% *W*_max_, 149.60° ± 31.52° versus 79.23 ± 29.99° s^−1^, *p* < 0.0001; and 60% *W*_max_, 204.35° ± 68.76° versus 110.15° ± 42.28° s^−1^, *p* < 0.0001).

#### Isometric knee extension exercise

##### Systolic

Similar to the cycling, during the isometric knee extension, apical rotation significantly increased from rest to 40% MVC then remained unchanged. Basal rotation demonstrated a trend towards reduced rotation from rest to 40% MVC (*p* = 0.07), then a trend towards an increase from 40 to 75% MVC (*p* = 0.05). LV twist remained unchanged throughout the incremental knee extension. Apical rotation velocity significantly increased from rest to 40% MVC then demonstrated a trend towards a further statistically significant increase to 75% MVC (*p* = 0.05), which was significantly different to rest. PTV progressively increased with exercise intensity from rest to 75% MVC, whilst basal rotation velocity only showed a significant change from rest to 75% MVC. Apical rotational parameters were significantly greater than the corresponding basal parameters at all intensities (Fig. 2b).

##### Diastolic

The isometric knee extension elicited a significant increase in diastolic apical rotation velocity progressively from rest to 75% MVC. Basal rotation velocity showed no changes between intensities and PUV did not elicit any changes from rest to 40% MVC, but was significantly greater at 75% MVC compared to rest and 40% MVC. As with the cycling, time-to-peak untwisting velocity remained unchanged throughout the protocol. PUV occurred significantly later into diastole, expressed as a %diastole from rest to 40% MVC then remained unchanged thereafter. During all intensities of the incremental isometric knee extension apical untwisting velocity was greater than basal untwisting velocity (rest, 65.77° ± 21.94° versus 47.60° ± 21.9° s^−1^, *p* < 0.01; 40% MVC, 92.30° ± 35.13° versus 49.21° ± 18.12° s^−1^, *p* < 0.0001; 75% MVC, 119.03 (66.78)° s^−1^ versus 62.70 (28)° s^−1^, *p* < 0.001).

## Discussion

The present study is the first to investigate the influence of incremental cycling and isometric knee extension exercises on LV twist mechanics in the same cohort. Furthermore, previously no systolic and diastolic twist mechanics data during intensities greater than 40% MVC were available. Accordingly, the main findings of this investigation are that, (1) LV twist increased from rest to 30% *W*_max_ then remained unchanged from 30 to 60% *W*_max_ during cycling, whilst systolic and diastolic twisting velocities progressively increased. (2) A novel finding is that short duration, incremental isometric knee extension preserved twist but elicited increases in systolic and diastolic twisting velocities. (3) During both cycling and isometric knee extension exercises, rotation and rotational velocity at the apex was significantly greater and faster than at the base, respectively.

### LV twist mechanics during incremental semi-supine cycling

#### Absolute systolic twist

Findings that systolic twist initially increased from rest to submaximal exercise concur with past work (Doucende et al. 2010; Lee et al. 2012; Stohr et al. 2012). Systolic rotations then remained unchanged following a further increase in intensity, suggesting a plateau in the ‘absolute’ magnitude of net rotation at moderate intensity (between 30 and 60% *W*_max_) and agree with our first hypothesis. In addition, these observations support the findings of the only previous investigation to use intensities greater than 40% peak power (Stohr et al. 2011). Mechanical constraints of the LV could be limited by multiple factors which may have profound implications on stroke volume (Stohr et al. 2011), including (1) pericardial constraints preventing further ventricular distension, (2) maximum systolic tissue compression, (3) increased blood pressure given the known afterload-twist interaction (Dong et al. 1999), and (4) an upper limit in titin phosphorylation. Acute phosphorylation during dynamic exercise may contribute to the Frank-Starling mechanism (Muller et al. 2014); therefore, an upper limit of phosphorylation may effectively limit twist, at least in recreationally active individuals. A combination of these factors may (in part) reflect an upper physiological limit to twist. Further study in elite endurance athletes may establish potential adaptability since athletes demonstrate reduced twist mechanics than untrained counterparts, suggestive of a functional reserve (Nottin et al. 2008; Santoro et al. 2014a).

Peak rotational mechanics were greater at the apex than base at rest and all cycling intensities. This is unsurprising since apical rotation is dominant in global LV twist (Notomi et al. 2006) and the apex demonstrates a more dynamic behaviour than the base (Doucende et al. 2010; Stohr et al. 2011) because of the LV geometric structure (Stöhr et al. 2015) and greater β-adrenergic receptor density (Mori et al. 1993).

#### Systolic and diastolic twisting velocities

Unlike absolute twist, PTV and PUV increased at both 30 and 60% *W*_max_ and further increases at higher intensities must be considered. Similar to this study PTV and PUV initially increased, but did not increase beyond moderate intensities indicating a potential upper limit (Stohr et al. 2011). The transition from rest to exercise diminishes the total cardiac cycle duration, most notably diastole, consequent to elevated HR (Doucende et al. 2010). As a result, faster myocardial rotation may be required during systole to accommodate these reductions (Doucende et al. 2010) and generate sufficient energy for release during early diastole to increase the intraventricular pressure gradient and augment LV filling during exertion (Burns et al. 2009). Although systolic twist and PUV are positively associated (Notomi et al. 2006), PTV may better predict PUV rather than the absolute twist as systolic and diastolic velocities were paralleled, expressing a systolic-diastolic coupling during incremental exercise in this study.

### LV twist mechanics during incremental isometric knee extension

#### Absolute systolic twist

To our knowledge this is the first study examining twist mechanics during lower body isometric exercise, showing preserved LV twist at both intensities, which is discordant with prior IHG work (Balmain et al. 2016; Weiner et al. 2012) and opposes our second hypothesis. An explanation for our findings is a potential lack of afterload effect. Despite progressively increased arterial blood pressure, at the same relative intensity (40% MVC) SBP and DBP were lower than those reported previously (Balmain et al. 2016; Weiner et al. 2012). Sustained IHG may have had a greater effect on afterload, explaining the impairment evident following 3 min of IHG in those studies compared to the preserved twist presented here. Thus, this suggests short duration knee extension may not have elicited sufficient afterload to influence twist. At 40% MVC enhanced cardiac output and MAP is solely driven by tachycardia via vagal withdrawal, whilst sympathetic activation only assists HR after 10 s at 75% MVC (Maciel et al. 1987). Given the short duration (15 s) of effort in this study, and the biphasic blood pressure response to isometric exercise, sympathetic activation could have further increased MAP towards the latter end of contraction. However, the relative contribution of this sympathetic drive is likely to be minimal relative to vagal withdrawal. Previous work has demonstrated that total peripheral resistance remains unchanged with short duration (20 s) IHG at progressively higher intensities despite increased muscle sympathetic nerve activity. Therefore, in short duration isometric exercise, increased MAP is predominantly a consequence of augmented HR, and thus cardiac output (Lalande et al. 2014). It is plausible that longer contractions, such as the 3 min IHG utilised previously (Balmain et al. 2016; Weiner et al. 2012), may accentuate afterload and explain the reduced LV twist in those studies. The implications of such reductions remain incompletely understood. However, chronic hypertensive and aortic stenosis patients showed increased baseline twist (Santoro et al. 2014b), which may be demonstrative of a compensatory mechanism to maintain normal cardiac function. Thus, it is clear that greater understanding surrounding how afterload inducing exercise effects acute twist mechanics may have important implications for chronic health and disease. A second explanation concerns a limited vascular response during the isometric exercise. Twist decreased after partial and full arm arterial occlusion (Balmain et al. 2016; van Mil et al. 2016), indicating that twist may be more sensitive to peripheral resistance and/or vascular-mediated rather than flow-mediated (cardiac output) blood pressure changes (Balmain et al. 2016), even within a normal afterload range and independent of HR and ischemia (van Mil et al. 2016). Indeed, LV twist and systemic vascular resistance are negatively associated (van Mil et al. 2016). In conjunction with the aforementioned maintenance of peripheral resistance during short duration IHG (Lalande et al. 2014) and although we observed rapidly increased MAP, an insufficient vascular response may have prevented changes in twist. It may be possible that either a lack of afterload or vascular effect or a combination of both were responsible for the preserved twist in this study. Nevertheless, this study is unique in that it is the first to investigate LV twist mechanics during isometric exercise utilising a large muscle mass. It would be beneficial for future work to replicate exercise training and explore the influence of multiple, consecutive repetitions and/or longer duration lower body isometric efforts on twist mechanics. The exercise pressor response would likely be larger and potentially evoke differing cardiac responses than observed in our study.

#### Systolic and diastolic twisting velocities

PTV progressively increased with incremental exercise, whereas PUV remained unchanged from rest to 40% MVC which agrees with previous IHG work (Balmain et al. 2016; Weiner et al. 2012). Our study extends these observations showing significantly increased PUV to 75% MVC. The magnitude and rate of untwisting are increased with pharmacological inotropic interventions (Rademakers et al. 1992). However, increased PUV at 75% MVC is likely due to predominantly greater withdrawal of vagal tone, as this is the primary mechanism for tachycardia at the initiation of isometric contractions (Maciel et al. 1987). Full circulatory occlusion reduced HR via vagal tone restoration, resulting in decreased PUV which indicates a HR response may confound the ‘true’ effect of arterial pressure on PUV (Balmain et al. 2016). Thus, it is plausible that limited increases in arterial pressure and sympathetic activation, the increased twisting mechanics as shown in this study be mediated solely by accentuated chronotropic effect. Similar to the aforementioned reduced systolic and diastolic durations, altered twisting velocities as opposed to absolute twist, may contribute a pivotal role in supporting LV function during such physiological conditions.

### Uncoupling of twist and twisting velocity: a comparison between dynamic and isometric exercise

Acute increases in HR during dynamic exercise results from both parasympathetic withdrawal and sympathetic activation, whereas tachycardia during short duration isometric exercise is predominantly due to vagal withdrawal in the absence of inotropic stimulus (Maciel et al. 1987). These different physiological responses to dynamic and isometric exercise may explain the observed LV twist mechanics of the present study. Cycling at 30% *W*_max_ and knee extension at 75% MVC indicates similar HR and afterload conditions (Table 3). Given that twist increased during cycling but remained unchanged during isometric exercise it is plausible that in dynamic exercise, absolute twist is driven by sympathetic activation, the greater preload or a combination of both. Moreover, the enhanced twisting velocities seen during both cycling and at 75% MVC may be mediated by changes in HR independent of sympathetic activity, at least at ~100 beats min^−1^. Further, post hoc analysis found PUV was greater at 30% *W*_max_ than 75% MVC (analysis not presented). Greater cardiovascular demand during dynamic exercise may account for this difference, through increases in either preload or higher inotropic effect following larger increases in noradrenaline, which were observed during equated static and dynamic exercise with similar HR (Lewis et al. 1985). Thus, it is postulated that additional preload or sympathetic activity may further accentuate twisting velocities secondary to HR. However, we acknowledge a comparison between dynamic and static exercise is difficult, based on the differing modalities and ensuing physiological responses (Zouhal et al. 2008).

Nevertheless, although both twist and twist velocity refer to the same physiological phenomenon regarding myocardial rotational mechanics, this unexpected observation of an uncoupling suggests their roles in enhancing LV functioning can occur independently of each other and potentially be facilitated by different physiological responses. Still, the present study was an exploratory study and future work would benefit from including the assessment of the autonomic nervous system to enable exploration of the interaction between autonomic nervous system and LV twist mechanics.

### Study limitations

Several limitations from this study warrant mention. We studied only the healthy young men so the responses observed during this investigation can only be generalised to this specific population. In addition to blood pressure, afterload is often confirmed with increased end-systolic volumes (Weiner et al. 2012); exercising volumes were not measured during isometric exercise because the focus was the observation of twist mechanics during short duration exercise with minimal repetitions. Moreover, although participants were tested at the same time of day and the influence of physical activity and caffeine were minimised, a large magnitude of variables can influence acute cardiac function, including but not extending to psychological stress, sleep quality, physiological stress, and hydration etcetera. A purpose of this study was to study LV twist mechanics during short duration isometric exercise, however, by doing so may have enhanced potential error in blood pressure measurements due to the rapid drop in pressure necessary to obtain both SBP and DBP within 15 s. The lead investigator performed and analysed all collected echocardiographic images so was therefore not blinded to the exercise conditions. We speculate a potential interaction between the autonomic nervous system and LV twist mechanics; however, we are unable to quantifiably evidence these suggestions as we did not measure components of the autonomic nervous system. However, as mentioned previously, future studies may wish to consider the autonomic system when studying LV twist mechanics during exercise.

In regards to technological confines, although the use of STE is considered a feasible approach to determine twist mechanics (Weiner et al. 2010a), it is not without its limitations. Large intra-observer variances are associated with twist mechanics (Burns et al. 2009), in particular apical rotation (Stewart et al. 2016) which is poorly defined and given its influence on twist and untwist presents technological issues. The precise basal and apical imaging planes may show intra-subject variability (Doucende et al. 2010) and especially during exercise, repeatable apical image acquisition along a consistent plane is challenging. The difficulty of echocardiography during exercise also presents clear implications for assessment inherent to using natural methods of inducing physiological stress.

## Conclusion

Results from this study demonstrate LV twist initially increases with semi-supine cycling then reaches an upper limit at moderate intensities, whereas systolic and diastolic twisting velocities progressively increase with incremental exercise. In addition, a novel finding is that a short duration, incremental isometric knee extension exercise does not impair twist mechanics. Further, despite unaffected systolic twist, twisting velocities appear to augment during lower body isometric exercise.

## Abbreviations

ANOVA: Analysis of variance
DBP: Diastolic blood pressure
HR: Heart rate
IHG: Isometric hand grip
LV: Left ventricle
MAP: Mean arterial pressure
MVC: Maximal voluntary contraction
PTV: Peak twisting velocity
PUV: Peak untwisting velocity
SBP: Systolic blood pressure
STE: Speckle tracking echocardiography
${\dot{V}\text{O}}_{{2\;{\text{peak}}}}$: Peak oxygen consumption
*W*_max_: Work rate maximum

## References

[CR1] Adler Y, Fisman EZ, Koren-Morag N, Tanne D, Shemesh J, Lasry E, Tenenbaum A (2008). Left ventricular diastolic function in trained male weight lifters at rest and during isometric exercise. Am J Cardiol.

[CR2] Arts FJ, Kuipers H, Jeukendrup AE, Saris WH (1993). A short cycle ergometer test to predict maximal workload and maximal oxygen uptake. Int J Sports Med.

[CR3] Balmain B, Stewart GM, Yamada A, Chan J, Haseler LJ, Sabapathy S (2016). The impact of an experimentally induced increase in arterial blood pressure on left ventricular twist mechanics. Exp Physiol.

[CR4] Biswas M (2013). Two- and three-dimensional speckle tracking echocardiography: clinical applications and future directions. Echocardiography.

[CR5] Bojsen-Moller J, Magnusson SP, Rasmussen LR, Kjaer M, Aagaard P (2005). Muscle performance during maximal isometric and dynamic contractions is influenced by the stiffness of the tendinous structures. J Appl Physiol (Bethesda, Md: 1985).

[CR6] Burns AT, La Gerche A, Prior DL, Macisaac AI (2009). Left ventricular untwisting is an important determinant of early diastolic function. JACC Cardiovasc Imaging.

[CR7] Burns AT, La Gerche A, Prior DL, Macisaac AI (2010). Left ventricular torsion parameters are affected by acute changes in load. Echocardiography.

[CR8] Cooke CB (2009). Maximal oxygen uptake, economy and efficiency.

[CR9] Donal E, Rozoy T, Kervio G, Schnell F, Mabo P, Carre F (2011). Comparison of the heart function adaptation in trained and sedentary men after 50 and before 35 years of age. Am J Cardiol.

[CR10] Dong SJ, Hees PS, Huang WM, Buffer SA, Weiss JL, Shapiro EP (1999). Independent effects of preload, afterload, and contractility on left ventricular torsion. Am J Physiol.

[CR11] Doucende G, Schuster I, Rupp T, Startun A, Dauzat M, Obert P, Nottin S (2010). Kinetics of left ventricular strains and torsion during incremental exercise in healthy subjects: the key role of torsional mechanics for systolic-diastolic coupling. Circ Cardiovasc Imaging.

[CR12] Drury CT, Bredin SS, Phillips AA, Warburton DE (2012). Left ventricular twisting mechanics and exercise in healthy individuals: a systematic review. Open Access J Sports Med.

[CR13] Gibbons Kroeker CA, Tyberg JV, Beyar R (1995). Effects of load manipulations, heart rate, and contractility on left ventricular apical rotation. An experimental study in anesthetized dogs. Circulation.

[CR14] Kong PW, van Haselern J (2010). Revisiting the influence of hip and knee angles on quadriceps excitation measured by surface electromyography. Int Sport Med J.

[CR15] Kuipers H, Verstappen FT, Keizer HA, Geurten P, van Kranenburg G (1985). Variability of aerobic performance in the laboratory and its physiologic correlates. Int J Sports Med.

[CR16] Lalande S, Sawicki CP, Baker JR, Shoemaker JK (2014). Effect of age on the hemodynamic and sympathetic responses at the onset of isometric handgrip exercise. J Appl Physiol (Bethesda, Md: 1985).

[CR17] Lang RM (2015). Recommendations for cardiac chamber quantification by echocardiography in adults: an update from the American Society of Echocardiography and the European Association of Cardiovascular Imaging. Eur Heart J Cardiovasc Imaging.

[CR18] Lee LS, Mariani JA, Sasson Z, Goodman JM (2012). Exercise with a twist: left ventricular twist and recoil in healthy young and middle-aged men, and middle-aged endurance-trained men. J Am Soc Echocardiogr.

[CR19] Lewis SF, Snell PG, Taylor WF, Hamra M, Graham RM, Pettinger WA, Blomqvist CG (1985). Role of muscle mass and mode of contraction in circulatory responses to exercise. J Appl Physiol (Bethesda, Md: 1985).

[CR20] Lower R (1669). Tracatus de corde.

[CR21] MacDougall JD, McKelvie RS, Moroz DE, Sale DG, McCartney N, Buick F (1992). Factors affecting blood pressure during heavy weight lifting and static contractions. J Appl Physiol (Bethesda, Md: 1985).

[CR22] Maciel B, Gallo L, Neto JM, Martins L (1987). Autonomic nervous control of the heart rate during isometric exercise in normal man. Pflüg Arch.

[CR23] Marginson V, Eston R (2001). The relationship between torque and joint angle during knee extension in boys and men. J Sports Sci.

[CR24] Mor-Avi V (2011). Current and evolving echocardiographic techniques for the quantitative evaluation of cardiac mechanics: ASE/EAE consensus statement on methodology and indications endorsed by the Japanese Society of Echocardiography. J Am Soc Echocardiogr.

[CR25] Mori H, Ishikawa S, Kojima S, Hayashi J, Watanabe Y, Hoffman JI, Okino H (1993). Increased responsiveness of left ventricular apical myocardium to adrenergic stimuli. Cardiovasc Res.

[CR26] Mosteller RD (1987). Simplified calculation of body-surface area. N Engl J Med.

[CR27] Muller AE, Kreiner M, Kotter S, Lassak P, Bloch W, Suhr F, Kruger M (2014). Acute exercise modifies titin phosphorylation and increases cardiac myofilament stiffness. Front Physiol.

[CR28] Nagueh SF (2009). Recommendations for the evaluation of left ventricular diastolic function by echocardiography. Eur Heart J Cardiovasc Imaging.

[CR29] Notomi Y (2006). Enhanced ventricular untwisting during exercise: a mechanistic manifestation of elastic recoil described by Doppler tissue imaging. Circulation.

[CR30] Notomi Y (2008). Ventricular untwisting: a temporal link between left ventricular relaxation and suction. Am J Physiol Heart Circ Physiol.

[CR31] Nottin S, Doucende G, Schuster-Beck I, Dauzat M, Obert P (2008). Alteration in left ventricular normal and shear strains evaluated by 2D-strain echocardiography in the athlete’s heart. J Physiol Lond.

[CR32] Rademakers FE, Buchalter MB, Rogers WJ, Zerhouni EA, Weisfeldt ML, Weiss JL, Shapiro EP (1992). Dissociation between left ventricular untwisting and filling. Accentuation by catecholamines. Circulation.

[CR33] Santoro A, Alvino F, Antonelli G, Caputo M, Padeletti M, Lisi M, Mondillo S (2014). Endurance and strength athlete’s heart: analysis of myocardial deformation by speckle tracking echocardiography. J Cardiovasc Ultrasound.

[CR34] Santoro A, Alvino F, Antonelli G, Zaca V, Benincasa S, Lunghetti S, Mondillo S (2014). Left ventricular twisting modifications in patients with left ventricular concentric hypertrophy at increasing after-load conditions. Echocardiography.

[CR35] Sengupta PP, Tajik AJ, Chandrasekaran K, Khandheria BK (2008). Twist mechanics of the left ventricle: principles and application. JACC Cardiovasc Imaging.

[CR36] Smolander J, Aminoff T, Korhonen I, Tervo M, Shen N, Korhonen O, Louhevaara V (1998). Heart rate and blood pressure responses to isometric exercise in young and older men. Eur J Appl Physiol Occup Physiol.

[CR37] Stefani L, Toncelli L, Di Tante V, Vono MC, Cappelli B, Pedrizzetti G, Galanti G (2008). Supernormal functional reserve of apical segments in elite soccer players: an ultrasound speckle tracking handgrip stress study. Cardiovasc Ultrasound.

[CR38] Stewart GM, Yamada A, Kavanagh JJ, Haseler LJ, Chan J, Sabapathy S (2016). Reproducibility of echocardiograph-derived multilevel left ventricular apical twist mechanics. Echocardiography.

[CR39] Stohr EJ, Gonzalez-Alonso J, Shave R (2011). Left ventricular mechanical limitations to stroke volume in healthy humans during incremental exercise. Am J Physiol Heart Circ Physiol.

[CR40] Stohr EJ (2012). Left ventricular mechanics in humans with high aerobic fitness: adaptation independent of structural remodelling, arterial haemodynamics and heart rate. J Physiol.

[CR41] Stohr EJ, Shave RE, Baggish AL, Weiner RB (2016). Left ventricular twist mechanics in the context of normal physiology and cardiovascular disease: a review of studies using speckle tracking echocardiography. Am J Physiol Heart Circ Physiol.

[CR42] Stöhr EJ, Stembridge M, Esformes JI (2015). In vivo human cardiac shortening and lengthening velocity is region dependent and not coupled with heart rate: ‘longitudinal’ strain rate markedly underestimates apical contribution. Exp Physiol.

[CR43] Streeter DD, Spotnitz HM, Patel DP, Ross J, Sonnenblick EH (1969). Fiber orientation in the canine left ventricle during diastole and systole. Circ Res.

[CR44] van Dalen BM, Vletter WB, Soliman OI, Folkert J, Geleijnse ML (2008). Importance of transducer position in the assessment of apical rotation by speckle tracking echocardiography. J Am Soc Echocardiogr.

[CR45] van Mil AC, Pearson J, Drane AL, Cockcroft JR, McDonnell BJ, Stohr EJ (2016). Interaction between left ventricular twist mechanics and arterial haemodynamics during localised, non-metabolic hyperaemia with and without blood flow restriction. Exp Physiol.

[CR46] Weiner RB (2010). The impact of endurance exercise training on left ventricular torsion. JACC Cardiovasc Imaging.

[CR47] Weiner RB (2010). Preload dependency of left ventricular torsion: the impact of normal saline infusion. Circ Cardiovasc Imaging.

[CR48] Weiner RB, Weyman AE, Kim JH, Wang TJ, Picard MH, Baggish AL (2012). The impact of isometric handgrip testing on left ventricular twist mechanics. J Physiol.

[CR49] Weippert M, Behrens K, Rieger A, Stoll R, Kreuzfeld S (2013). Heart rate variability and blood pressure during dynamic and static exercise at similar heart rate levels. PLoS One.

[CR50] Zouhal H, Jacob C, Delamarche P, Gratas-Delamarche A (2008). Catecholamines and the effects of exercise, training and gender. Sports Med.

